# Cockayne syndrome mutation in *XPG* activate the integrated stress response

**DOI:** 10.1007/s00439-025-02804-3

**Published:** 2026-01-21

**Authors:** Danhui Zhang, Max Hartmann, Zhouli Cao, Gaojie Zhu, Gregoire Najjar, Cagatay Günes, Steffen Emmert, Karin Scharffetter-Kochanek, Sebastian Iben

**Affiliations:** 1 Department of Dermatology and Allergic Diseases, Ulm University, 89081 Ulm, Germany; 2 Department of Urology, Ulm University, 89081 Ulm, Germany; 3 Clinic and Policlinic for Dermatology and Venerology, University Medical Center Rostock, 18051 Rostock, Germany

## Abstract

Xeroderma pigmentosum (XP) and Cockayne syndrome (CS) are diseases provoked by mutations in multifunctional proteins that are involved in DNA repair. DNA-repair deficiency explains the high cancer incidence of XP, whereas cancer-free CS, characterized by growth retardation, neurological degeneration, and premature aging does not present as a classical DNA-repair deficiency disorder. Here, we compared a severe combined XP/CS case provoked by *XPG*-mutation with an XP “only” patient cell line caused by mutation in the same *XPG* gene to carve out the pathogenic cellular disturbances that provoke CS. We identified RNA polymerase I transcription and rRNA maturation defects, a highly phosphorylated eukaryotic initiation factor 2 alpha (eIF2alpha), and a shift from cap- to internal ribosomal entry site (IRES) translation, indicating an activated integrated stress response in CS. Disturbances in ribosomal biogenesis and translational control might thus contribute to the development of CS.

**Supplementary Information:**

The online version contains supplementary material available at 10.1007/s00439-025-02804-3.

## Introduction

Nucleotide-excision-repair (NER) is a central DNA-repair pathway essential for the removal of helix-distorting DNA-lesions and involved in the repair of oxidative DNA-damage (Kuper and Kisker 2023) NER is in the first line of defense against the mutagenic properties of ultraviolet (UV) light, and in fact, mutations in NER-factors provoke the high skin cancer-prone disease xeroderma pigmentosum (XP). XP is characterized by dry and hyperpigmented skin, and the “moonshine” children, who need to be shielded from sunlight, suffer from a 2000–10.000 fold elevated risk of getting all types of skin cancer after UV-exposure (Kraemer et al. 1987). Mutations in five excision-repair cross-complementation (*ERCC*) group genes, also called *XPA- XPG* as well as the XP variant type, are causal for XP and some gene mutations are causing adult-onset neurological degeneration (Kraemer et al. 1993). Mutations in NER factors can also provoke two childhood disorders of impaired development, signs of premature aging, severe neurodegeneration often leading to early death: Cockayne syndrome (CS) and trichothiodystrophy (TTD) (Ferri et al. 2020). Whereas in XP, cancer development can be prevented by UV-protection through special clothing, allowing normal development, childhood degeneration in CS and TTD cannot be prevented (Bukowska and Karwowski 2018). Therefore, the canonical DNA-repair function of NER, sheltering us from UV-induced cancer, cannot be the underlying pathomechanism in CS and TTD (Brooks 2013). As part of the NER proteins are also involved in basal transcription by RNA polymerases I and II (Assfalg et al. 2012; Bradsher et al. 2002; Koch et al. 2014), CS and TTD are discussed to be transcriptional syndromes that are caused by failures in basal gene expression (Egly 2001). In the case of TTD, general disturbances in ribosomal biogenesis by RNA polymerase I and in consequence disturbed ribosomal functions could be described in a broad variety of TTD-causing gene mutations (Khalid et al. 2023; Phan et al. 2021; Zhu et al. 2023), all leading to a loss of proteostasis, one hallmark of aging (Lopez-Otin et al. 2013, 2023). Also, in CS, a loss of proteostasis by a dysfunctional ribosomal biogenesis and performance has been reported for causal mutations in the NER factors cockayne syndrome protein A (CSA) and cockayne syndrome protein B (CSB) (Alupei et al. 2018; Qiang et al. 2021). Loss of proteostasis is a pathogenetic process in neurodegenerative diseases (Lopez-Otin et al. 2023). Therefore, it is tempting to speculate that the neurodevelopmental/neurodegenerative symptoms of CS and TTD could originate from a disturbed ribosomal biogenesis and function. The NER factor excision-repair cross-complementation group 5 (*ERCC 5*)/xeroderma pigmentosum complementation group G (*XPG*) is a 3´endonuclease that helps to excise the UV-damaged DNA strand and stabilizes the NER DNA-repair and basal transcription factor, transcription factor II H (TFIIH) (Pal et al. 2022). *XPG* mutations can either cause XP or XP/CS. The XPG protein can be immunoprecipitated with an RNA polymerase I transcription complex (Bradsher et al. 2002), and a recent report identified RNA polymerase I transcription to be disturbed in a severely affected XPG-patient with XP/CS (Taupelet et al. 2022). In this study, the authors described pre-ribosomal RNA (rRNA) maturation disturbances and undissolved R-loop formation at the ribosomal DNA (rDNA) locus as cellular defects in XP/CS. The aim of the current study was to compare different disease entities provoked by mutations in the same *XPG* gene. As a loss of proteostasis by the ribosome was previously identified by us as a pathomechanism in CSA and CSB mutant CS (Alupei et al. 2018; Qiang et al. 2021), we here investigated if this pathomechanism is also active in CS forms provoked by unrelated mutations. Here we show that *XPG* mutation leading to severe XP/CS, but not to XP, affects RNA polymerase I transcription and strongly impacts the phosphorylation of the eukaryotic initiation factor 2 alpha (eIF2alpha), thereby inhibiting overall cap-dependent translation and favoring internal ribosomal entry site (IRES) translation.

## Material and methods

### Cell culture

Healthy wild type controls (1306 and FF95) and patient cell lines (XPCS1RO, XP56BR, and XP118BR were cultured in DMEM (41965-039, Gibco) with 10% FBS (S0615, Sigma-Aldrich), 1% penicillin-treptomycin (P06-07100, Bio-Techne) and 2 mM L-glutamine (P04-80100, Bio-Techne). Detailed information about the SV40/hTERT transformed cell lines used in this study is shown in Supplemental Table 1. Cells were passaged when reaching around 80% confluency. All cells were cultured in the incubator under 5% CO_2_ and 3% O_2_ at 37 °C.

**Table 1:** Materials used in cDNA synthesis

Reagents	Sequence 5’–3’
RT primer	AAGCAGTGGTATCAACGCAGAGTACT(30)VN
Template Switching Oligo	AAGCAGTGGTATCAACGCAGAGTACGCrGrGrG
cDNA PCR primer	AAGCAGTGGTATCAACGCAGAGT

### Genomic DNA (gDNA) isolation

gDNA was extracted by using the Easy-DNA gDNA purification kit (K180000, Invitrogen) and following the manufacturer’s instructions. Cell pellets were dissolved in 320 μl TE buffer including 20 μl solution A, 10 μl solution B, and 5 μl proteinase K, and then shaken overnight at 60 ℃. After adding 300 μl solution A and 120 μl solution B, the samples were mixed sufficiently until uniformly viscous. 750 μl of chloroform was added, and then the upper aqueous phase (gDNA) was isolated after full-speed centrifugation for 20 min. DNA was purified and washed by using 100% and 80% pre-cold ethanol, respectively, and resuspended in nuclease-free water. gDNA was stored at 4 ℃ for further use.

### RNA extraction and complementary DNA (cDNA) synthesis

RNA was extracted by using the RNeasy mini kit (74106, QIAGEN). The concentration of RNA was measured by using Nanodrop. After extraction, 400 ng RNA was used for cDNA synthesis by using the Template Switching RT Enzyme Mix (#M0466, NEB) according to the manufacturer’s description. RT primer, Template Switching Oligo, and cDNA PCR primer used in this experiment are listed in Table 1.

### PCR and sequence analysis

After gDNA extraction and cDNA synthesis, DNA products were amplified by using Q5 high-fidelity 2 × master mix (M0492S, NEB). Briefly, DNA products were mixed with Q5 high-fidelity 2 × master mix (M0492S, NEB), 10 μM forward primers, and 10 μM reverse primers. The PCR reactions were performed as follows: 98 °C 30 s for initial denaturation, 98 °C 10 s for denaturation, 63°–69 °C (depending on the different types of primers) for 30 s of annealing, 72 °C 30 s of extension, 72 °C 10 min of final extension. PCR products were harvested after 35 cycles. Used primers are listed in Supplemental Table 2 (for cDNA) and Supplemental Table 3 (for gDNA). PCR products were run on a 0.9% agarose gel, and targeted DNA fragments were cut under UV light. DNA products were extracted by using the gel extraction kit (28704, QIAGEN) and then sent for Sanger sequencing. Results were analyzed by using the CLC Genomics Workbench 22.0 software.

### RNA isolation and qRT-PCR

RNA was extracted by using the RNeasy mini kit (74,106, QIAGEN) according to the manufacturer’s instructions. The concentration of eluted RNA was measured by Nanodrop. 1 µg RNA was incubated with 250 ng random hexamer primer p(dN)6 (10558621, Roche GmbH) for 5 min at 70 °C. After binding with p(dN)6, RNA was reverse transcribed under M-MLV reverse transcriptase catalytic action by adding reaction mix (10 mM dNTP (U151B, Promega GmbH) 0.5 µl, 40U/µl RNase inhibitor (N2518, Promega GmbH) 0.5 µl, 200 U/µl M-MLV reverse transcriptase (M170B, Promega) 1 µl, and M-MLV 5 × Buffer (M531A, Promega GmbH) 4 µl) and incubating at 37 ℃ for 1 h. Synthesized cDNA was diluted to 1:50 before qPCR. 5 µl diluted cDNA mixed with Sybr-green 2*qPCR master mix (4913914001, Roche) and primers. After 40 cycles of PCR reaction, the Ct values of targeted gene expression were detected. The expression of *XPG*, pre-rRNAs, and genes involved in ER stress was performed using the QuantStudio™ 5 real-time PCR system. Results were analyzed by using QuantStudio™ design & analysis v1.5.2 software. Data was normalized to the level of β-actin. Primers used for qPCR experiments are listed in the Supplementary Table 4.

### Western blot assay

The cell pellets were resuspended in lysis buffer with protease and phosphatase inhibitor (78441, ThermoFisher), and the harvested protein concentration was measured by Bradford (5000006, BioRad). 20–50 μg total protein was loaded on an SDS-PAGE gel and was run at 80–120 V with 1 × MOPS–SDS running buffer. The gel was then transferred to a nitrocellulose membrane in the transfer buffer at 30 V overnight or 100 V for 1.5 h. The membrane was blocked for 1.5 h in 5% milk blocking solution (5% milk in TBST) at room temperature and then incubated with primary antibody (1:500–1:1000 diluted in milk solution) overnight at 4 °C. After washing with TBST, membranes were incubated with secondary antibody (1:10,000 diluted in milk solution) for 1.5 h at room temperature. After washing again with TBST 3 times, membranes were exposed under catalysis of chemiluminescent reactions (34580, ThermoFisher Scientific) and developed on Fusion Fx7 (Viber). Images were analyzed and quantified by Image J. Antibodies used for western blot experiments are listed in the Supplementary Table 5.

### Translational fidelity analysis

After being detached by accutase, cells were pelleted by centrifugation (100 g at 4 °C). Cell pellets were resuspended with medium, and cell concentration was measured by cell counter. 1.5 × 10^5^ cells were transfected with 5 μg Nano luciferase plasmid (positive control or mutant, or negative control) and 0.1 μg Firefly plasmid via electroporation by Neon™ transfection system with the following parameters: 1400 V 20ms, 1 pulse. Plasmids were used from Hartmann et al. (Hartmann et al. 2025). Transfected cells were seeded on 96 well-white plates and cultured for 24 h. Luciferase activity was measured by using the Dual-Luciferase Reporter Assay System (N1620, Promega) according to the manufacturer’s protocol. The ratio of Nano luciferase and Firefly luminescence was calculated and used as an indicator for translational fidelity.

### BisANS assay

BisANS dye is a probe for non-polar cavities in proteins and can detect the hydrophobic group; therefore, it is used as a protein folding and protein stability indicator. Cell pellets were resuspended in BisANS labeling buffer (50 mM Tris–HCl, 10 mM MgSO_4_, adjusted to PH 7.4) and then sonicated for 30 s 3 times. After centrifugation (16,900 *g*, 20 min), the supernatant was collected, and protein concentration was measured by Bradford protein assay. 100 μg of protein was incubated with 2 M urea solution for 2 h at room temperature. 30 μM BisANS dye (4,4’-dianilino-1,1’-binaphthyl-5,5’-disulfonic acid, dipotassium salt) was added to each sample and then transferred into a 96-well white bottom plate. Fluorescence was measured by VARIOSKANTM LUX with set parameters (excitation 375 nm and emission 500 nm).

### Heat sensitivity analysis

Heat sensitivity analysis was adapted from Treaster et al. (2014). Cell pellets were harvested and resuspended in 1.5 × packed cell volume (PCV) of Dignam A buffer (10 mM KCl, 10 mM Tris pH 7.9, 1.5 mM MgCl_2_, 1 mM DTT, 1:50 complete proteinase inhibitor mix) for 10 min on ice. Samples were swelled in this hypotonic buffer by passing through a 23G syringe 50–60 times. The lysates were centrifuged at 104,000 *g* for 20 min at 4 ℃. Supernatant was transferred to a new tube and centrifuged at 100,000 g for 1h at 4 ℃. 100–200 μg protein was heated at 99 ℃ for 15 min. After centrifugation (full speed, 5 min), the supernatant and the pellet were separated. Pellets were resolved in 4M urea. Then, the supernatant and pelleted protein concentrations were measured using the Bradford method. Finally, the percentage of pelleted protein in relation to total protein was calculated.

### Protein synthesis assay

Protein synthesis assay was performed by using the Protein Synthesis Assay Kit (601100, Cayman Chemical) according to the manufacturer’s description. In brief, 5 × 10^4^ cells were seeded in a blank 96-well plate. Cells were incubated with o-propargyl-puromycin (OPP) probe for around 1 h to label translating proteins. After that, the OPP-labeled proteins were detected by adding 5 FAM-Azide for 30 min. After 3 times washing, fluorescence was measured by VARIOSKANTM LUX under the excitation of 485nm and emission of 535nm.

### 8-OHdG ELISA

8-OHdG levels in all cell lines were measured by using the 8-hydroxy-2-deoxyguanosine ELISA Kit (ab201734, Abcam). gDNA extraction was performed as previously described. 30μg gDNA was digested by using 600 units of nuclease P1 (M0660s, New England Biolabs) according to the manufacturer’s instructions. Digested gDNA was dephosphorylated by incubating with 0.3 units of alkaline phosphatase (M0289S, New England Biolabs) at 37 °C for 30 min. Samples were boiled for 10 min to stop the reaction. All reagents in the ELISA kit were put at room temperature for at least 30 min before use. 50 μl of either prepared 8-hydroxy-2-deoxyguanosine standard or samples was added to a provided 96-well plate. 50 μl of diluted 8-OHdG antibody was added to each well and incubated for 1 h at room temperature. After 4 times of washing, 100 μl of TMB substrate was added to each well and covered with tin foil. 30 min later, the reaction was stopped by adding 100 μl of stop solution, and the color of the samples changed from blue to yellow. The absorbance of each well was measured at 450 nm using the VARIOSKANTM LUX instrument. A standard curve was generated, and the concentration of 8-OHdG in each sample was calculated based on the established standard curve.

### Carbonylation assay

The protein carbonylation level in each cell line was measured by using the Protein Carbonyl content assay kit (MAK094, Sigma). In brief, cell pellets were lysed in lysis buffer including protein inhibitor (1:50). 0.5–1.5 mg protein was used for further experiments. Samples were added with 10 μl 10% Streptozocin solution to remove nucleic acids. After centrifuging, the protein lysate was incubated with 100 μl 2,4-dinitrophenyl-hydrazine (DNPH) solution for 10 min. Proteins were precipitated by adding 30 μl of 87% TCA. After washing with ice-cold acetone three times to remove free DNPH, 200 μl 6M Guanidine solution was added to resolve the pelleted protein. The carbonylation level in each sample was measured at 375 nm by using the microplate absorbance reader. Carbonylated protein expression was normalized by total protein level (measured by BCA assay).

### ROS level detection

Dihydroethidium (DHE) (Invitrogen, D11347) was used to detect ROS levels in our cell lines. 5 × 10^5^ cells were incubated with 10 μM DHE for 15 min. After being washed once with FACS buffer (PBS including 1% FBS), cells were harvested in 200 μl FACS buffer. Fluorescence was detected by flow cytometry, and results were analyzed by FlowJo software (v10.8.1).

### Apoptosis assay

Apoptosis assay was performed by using FITC Annexin V apoptosis detection kit I (556547, BD Pharmingen). The same number of cells was seeded in 6-well dishes. After cells were attached, 2.5 mM potassium bromate in a novel medium was added to XPG/CS, XPG/XP, and control cell lines; the same volume of medium was added as a negative control. After incubating for 24 h, cells were collected and stained with Annexin V and propidium iodide (PI) for 15 min. Fluorescence was detected by flow cytometry and analyzed by FlowJo software (v10.8.1).

### T7 transcription

The cricket paralysis virus (Crpv)-IRES plasmid for T7 transcription was kindly provided by Dr. Marianna Penzo. T7 transcription was performed by using the HighYield T7 ARCA mRNA Synthesis Kit (RNT-102-S, Yena Bioscience). Briefly, 2 μl DTT (100 mM) was mixed well with 2 μl 10 × AmpliCap-Max T7 transcription buffer. After that, 500 ng linearized template DNA, 1.2 μl ANCA (100 mM), 1.5 μl dNTPs (100 mM), 2 µl 40 U/µl RNase inhibitor (N2518, Promega GmbH), and 2 µl T7 polymerase mix were added to the buffer. Samples were then incubated at 37 ℃ for 2 h in the dark. After removing all template DNA by using DNase I-XT (M0570S, New England Biolabs), RNA was purified via the RNeasy mini kit (74,106, QIAGEN). Eluted RNA was stored at -80℃ for further use.

### In vitro translation assay

In vitro translation experiments were performed by following the protocol of Penzo (2016). In vitro translation was performed until cells were around 70% confluence. Pellets were collected after digestion, then washed once with Dignam A buffer, and centrifuged at 100 *g* for 5 min. Cell pellets were resuspended in Dignam A buffer and incubated on ice for 10 min. Cells were swollen in Dignam A buffer by passing through a 23 G syringe 50–60 times. After being centrifuged at 10,400 g for 20 min, the supernatant was collected, and the protein concentration was measured by using Bradford. 600 ng CrPV-IRES RNA was added to the cell extract (around 40–70 μg protein). The components are listed in Supplementary Table 6. After 3 h of incubation at 30 ℃, firefly and renilla luminescence were measured by using VARIOSKANTM LUX.

### Statistics

All data were analyzed by using GraphPad Prism software (GraphPad 6). Each experiment was repeated at least 3 times. Statistical differences in two sample groups were assessed using the unpaired *t*-test (two-tailed), while comparisons among three or more sample groups were calculated using ANOVA. In the figures, asterisks (*) in the figures represent ρ values (* = *ρ* < 0.05, ** = *ρ* < 0.01, *** = *ρ* < 0.001) indicating the level of statistical significance.

## Results

### Mutation analysis and expression profiles of *XPG* in patient-derived cells

**Fig. 1:**
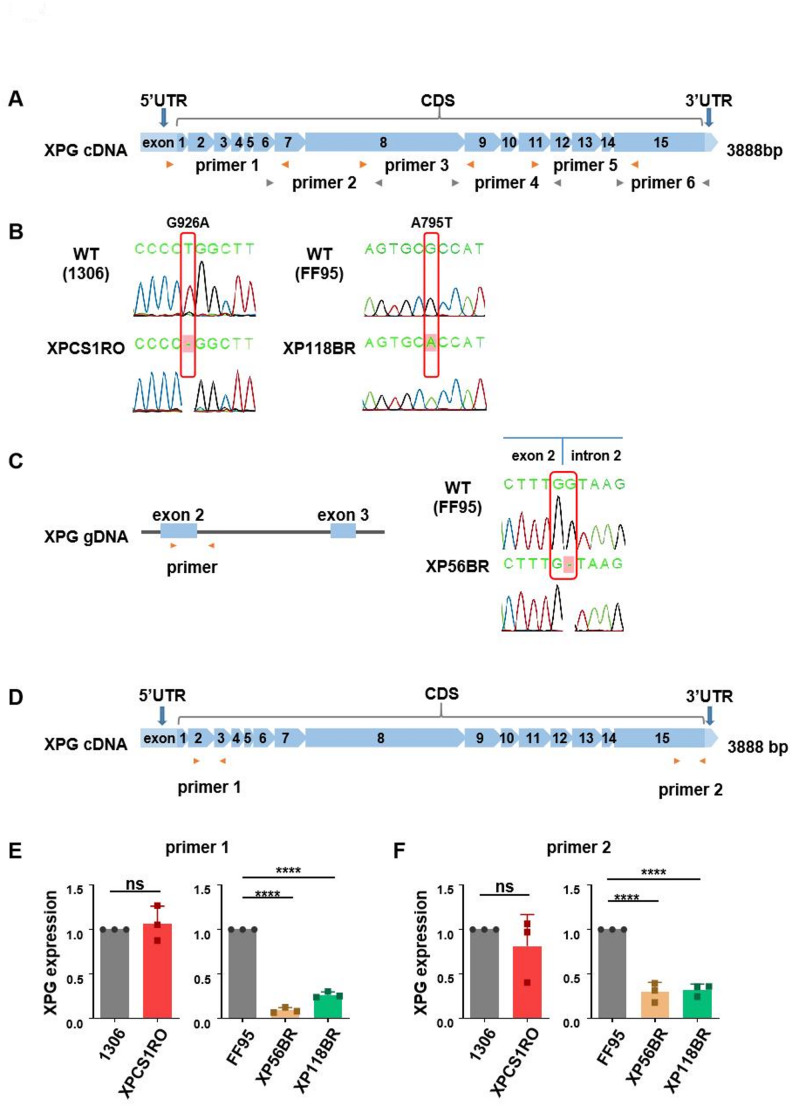
Cell line verification and *XPG* expression in wild type cell lines and patient cell lines. (**A**) Structural map of *XPG* cDNA and 6 pairs of primers used in sequence analysis. (**B**) Sequence analysis of XPCS1RO (left) and XP118BR (right) compared with healthy controls shows homozygous mutations (c.3010delT [p.G926AfsX56] and c.2618G > A [p.A795T] in XPCS1RO and XP118BR cell lines, respectively) (NM_000123.4). (**C**) Structural map of *XPG* gDNA and primers used for XP56BR cell line sequence (left). Sequence analysis of XP56BR compared with wild type shows a deletion of G at the boundary junction of exon 2 and intron 2 (right). (**D**)–(**F**) *XPG* mRNA expression in wild type cell lines and disease cell lines. (**D**) Schematic diagram of *XPG* cDNA and 2 pairs of primers used in qPCR. (**E**) and (**F**) Relative expression of *XPG* in wild type controls (1306 and FF95), severe CS (XPCS1RO), mild CS (XP56BR), and XP (XP118BR) cell lines; β-actin was used as an internal control for normalization. Data are represented as mean ± SD of at least three independent experiments. ns p > 0.05, * p ≤ 0.05, ** p ≤ 0.01, *** p ≤ 0.001, **** p ≤ 0.0001

Mutations in the *XPG* gene can give rise to different disease entities with varying severity. We chose to use a cell line from a Cockayne syndrome patient with early onset of symptoms and early infant death (XPCS1RO) (Hamel et al. 1996), a cell line from a mild CS case (XP56BR) and a cell line from an XP patient (XP118BR) (Fassihi et al. 2016) without CS symptoms. As controls, we used the SV40 transformed 1306, respectively, the hTERT transformed FF95 cells. In a first cell line verification step, we amplified with 6 primer pairs the *XPG* cDNA and performed Sanger sequencing (Fig. 1A). In fact, we were able to verify the reported homozygous deletion of one nucleotide in the XPCS1RO cell line, leading to a frameshift in the *XPG*-sequence and the homozygous base-exchange in the XP-cell line XP118BR (Fig. 1 B). The CS cell line from a mildly affected patient was found to bear a deletion at the border of exon 2 and intron 2, leading to a homozygous partial deletion of exon 1 and exon 2 (Fassihi et al. 2016) (Fig. 1C). Using qPCR and two primers against the 5´and 3´ends of the *XPG* mRNA of we revealed an *XPG*-mRNA expression at control levels in cells from the severe CS case (Fig. 1D–E), but a significantly reduced mRNA expression in cells of the mildly affected CS and XP patients. The two control cell lines 1306 and FF95 displayed three sequence variations to the reference sequence (NM_000123.4), two of them influencing the amino acid composition (Supplementary Fig. 2A) (Emmert et al. 2001). The predicted protein structure based on our sequencing result is schematically depicted in the graphs of Supplementary Fig. 2B. XPG stabilizes the transcription/DNA repair factor TFIIH (Ito et al. 2007; Iyer et al. 1996) and we monitored the abundance of TFIIH in these cell lines by Western blot. As shown in Supplementary Fig. 3, we could not detect a significant reduction of TFIIH in CS or XP cell lines, although TFIIH abundance seemed to correlate with XPG expression and is non-significant reduced in in the mild CS and XP cell line.

### Ribosomal biogenesis disturbances in the severe XPG/CS case

**Fig. 2:**
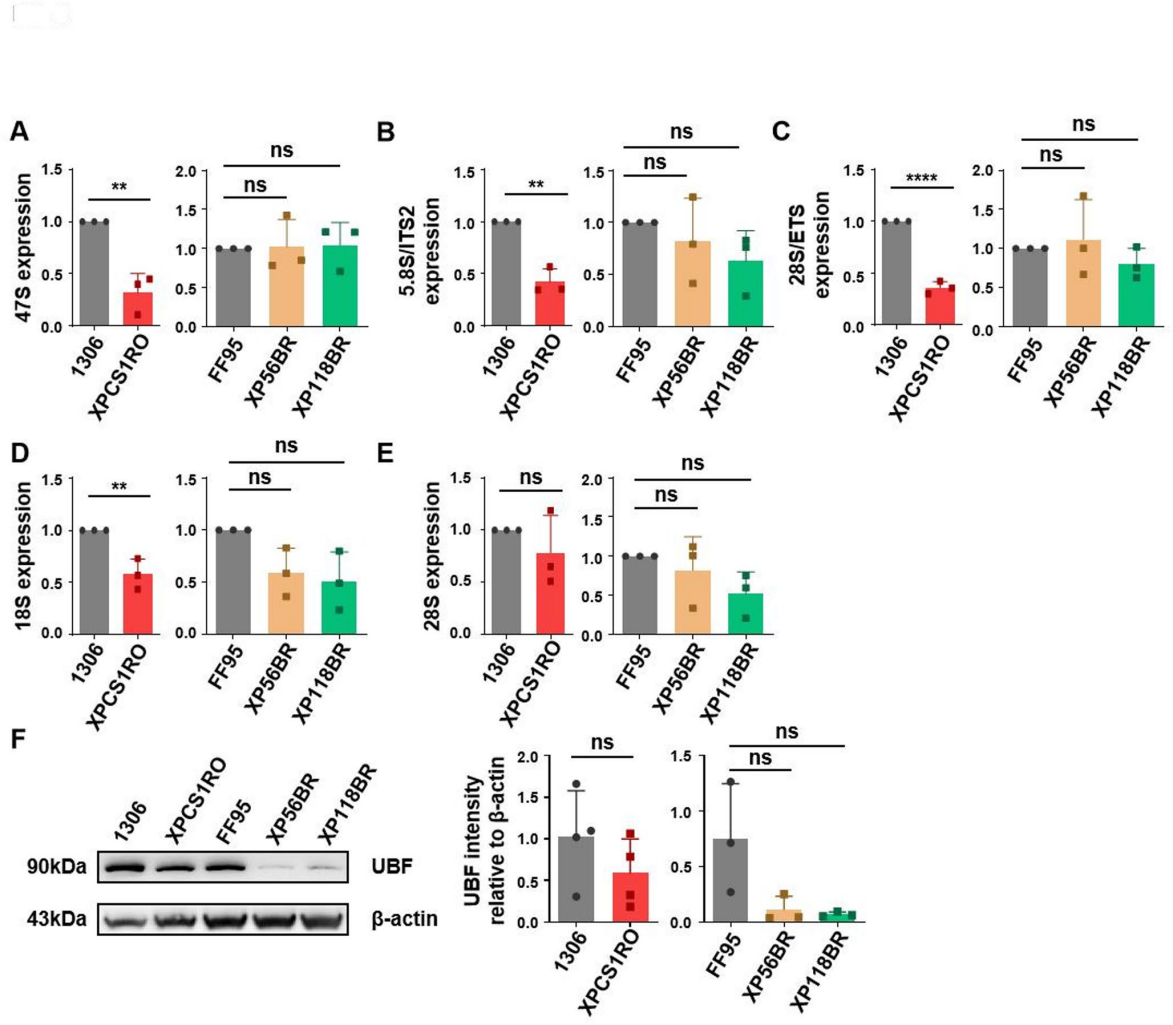
Pol I transcription in control cell lines and disease cell lines. (**A**)–(**E**) qPCR analysis of pre-rRNA (47S), rRNA transcription elongation (5.8S/ITS 2), termination (28S/ETS), and mature rRNA (18S and 28S) expression in control cell lines and *XPG* mutated cell lines. The values were normalized with β-actin. (**F**) Western blot analysis of UBF expression in control cell lines (1306 and FF95) and disease cell lines (XPCS1RO, XP56BR, and XP118BR) (left). Quantification of UBF expression in western blot (right). β-actin was used as an internal control. Data are represented as mean ± SD of at least three independent experiments. ns p > 0.05, * p ≤ 0.05, ** p ≤ 0.01, *** p ≤ 0.001, **** p ≤ 0.0001

In previous works, we could show that cells from CS patients with CSA/CSB or TFIIH mutations (Assfalg et al. 2012; Bradsher et al. 2002; Koch et al. 2014) are characterized by severe disturbances in RNA polymerase I transcription, the key step of ribosomal biogenesis. Therefore, we analyzed parameters of rDNA transcription and monitored the abundance of mature rRNA by qPCR. In fact, the cell line from the severe CS case displayed a reduction of the 47S pre-rRNA, which is rapidly cleaved and indicates transcription initiation (Fig. 2A). The abundance of gene-internal (5.8S/ITS2) and terminal 28S/ETS sequences is reduced in the severely affected CS patient cells, suggesting a reduced or repressed rRNA transcription elongation and termination (Fig. 2B–C). Moreover, the abundance of the functional and structural backbone of the small ribosomal 40S subunit, the mature 18S rRNA, is significantly reduced in the severe CS cell line (Fig. 2D), again resembling ribosomal abundance disturbances in other forms of CS (Qiang et al. 2021). The backbone of the 60S ribosomal subunit, 28S was not found to be affected (Fig. 2E). In line with these results is the qPCR analysis result of ITS1 abundance, a surrogate marker of pre-rRNA processing intermediates, that was also found to be reduced in the XPCS1RO cells (Supplementary Fig. 4). One central transcription factor of RNA polymerase I is the upstream binding factor (UBF) that we found reduced in the phenotypical mild CS and XP cell lines, but not in the severe CS cell line (Fig. 2F). This indicates that the disturbed RNA polymerase I transcription identified in these cells is not due to a reduced UBF abundance. Why the reduced UBF abundance in the mild CS and XP case does not impact on the measured ribosomal biogenesis parameters still needs further investigation. The non-significant reduced TFIIH content shown in supplemental Fig. 3 does not influence ribosomal biogenesis, indicating that mutation (Assfalg et al 2012), not abundance of TFIIH impacts on RNA polymerase I transcription.

**Fig. 3:**
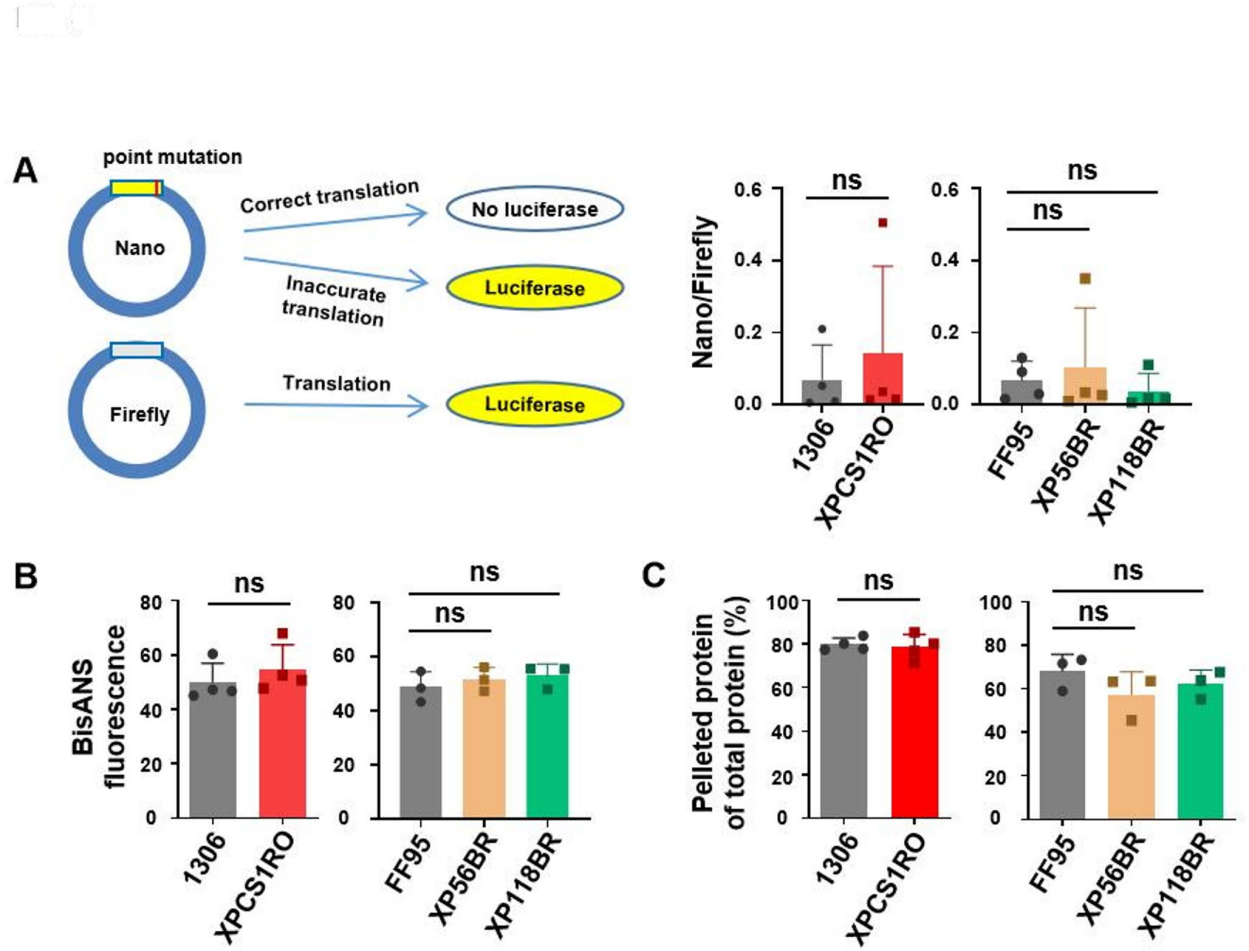
(**A**) Schematic diagram of translational fidelity assay. Mutated luciferase reporter (Nano luciferase) and firefly luciferase (transfection control) are co-transfected into cells. luciferase signal can be detected when ribosomes are error-prone (left). Translational fidelity is not affected by mutations in *XPG,* leading either to CS or XP (right). (**B**) Unfolded protein levels in control and disease cell lines were detected by BisANS fluorescence dye after being treated with 2M urea for 2 h. (**C**) Heat sensitivity analysis of cytoplasmic protein extracted from wild type cell lines and *XPG* mutated cell lines. Data are represented as mean ± SD of at least three independent experiments. ns p > 0.05, * p ≤ 0.05, ** p ≤ 0.01, *** p ≤ 0.001, **** p ≤ 0.0001

### Ribosomal accuracy and proteome stability are not affected in XPG/CS cells

The next series of experiments were inspired by previous results in CSA/CS and CSB/CS cells that display an elevated error rate of translation and an unstable proteome (Alupei et al. 2018; Qiang et al. 2021). We transfected a mutant luciferase-reporter plasmid into the different cells and monitored erroneous reactivation of luciferase activity by cellular translation. Interestingly and in sharp contrast to previous reports, we could not detect an elevated error rate of CS-cells (Fig. 3A). These results were in line with the proteome analyses-BisANS fluorescence measures exposed hydrophobic sites after unfolding stress by urea, and is not elevated in the assessed cell lines. A short heat treatment and subsequent centrifugation and quantification of proteins revealed that the proteome of CS cells is not aggregation-prone as previously shown for CSA and CSB cells (Fig. 3B, C). This suggests that on one side, proteome stability is linked to ribosomal fidelity, and on the other side, that proteome instability is not a general feature of CS.

### Integrated stress response (ISR) is highly activated in the severe XPG/CS case

**Fig. 4:**
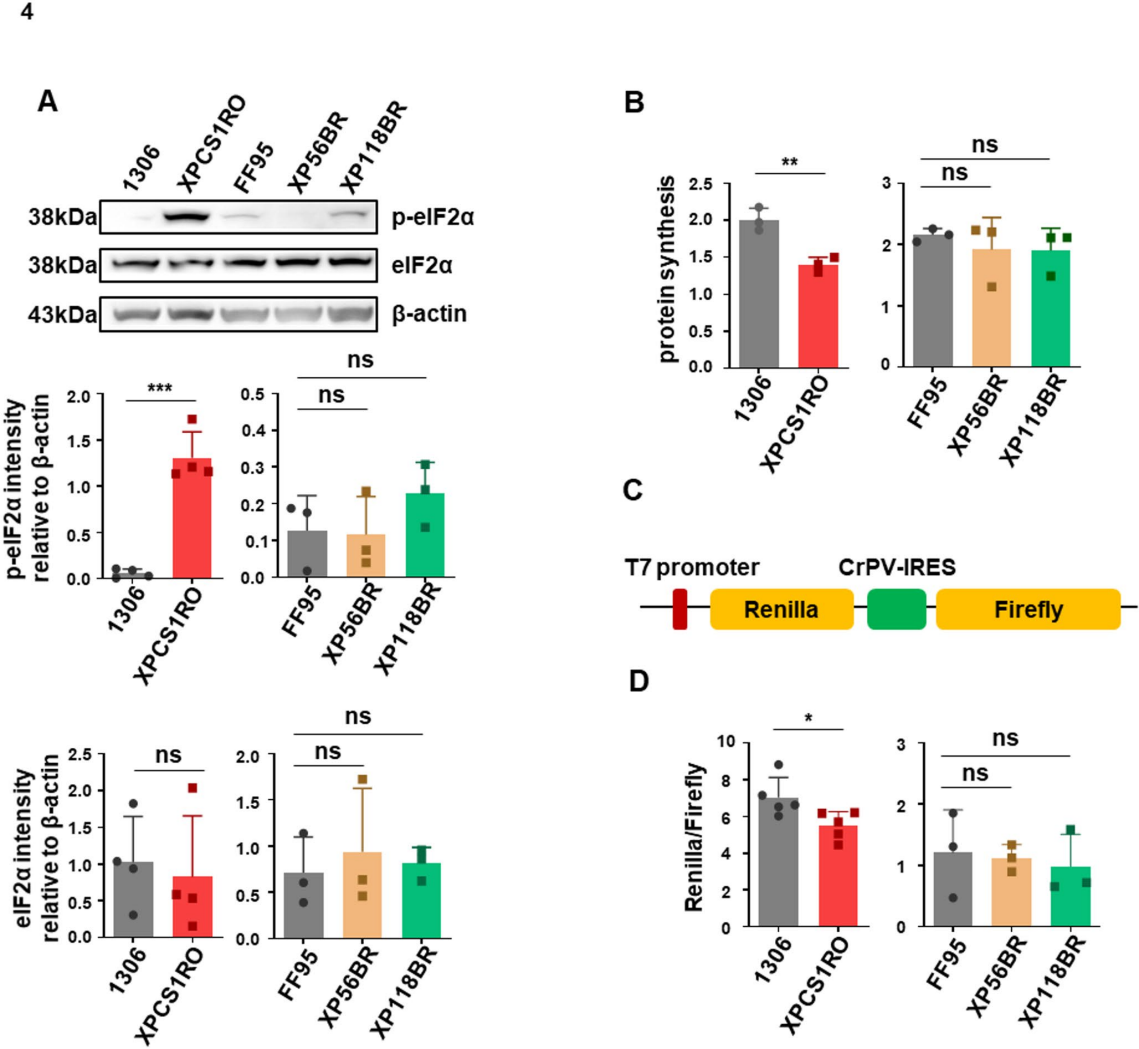
(**A**) Western blot analysis of p-eIF2α and eIF2α expression in control and disease cell lines. Protein abundance was normalized with β-actin. (**B**) Nascent synthesized protein was labeled by OPP in control and disease cell lines and detected by 5 FAM-Azide. (**C**), (**D**) In vitro translation of renilla luciferase and firefly luciferase. Diagram of mRNA used in in vitro translational assay. (**C**) Two yellow frames represent renilla luciferase and firefly luciferase sequences, respectively. The red area is the T7 promoter, and the green rectangle represents the CrPV-IRES sequence. The upstream CrPV-IRES sequence of the firefly luciferase gene leads to the translation initiation of firefly luciferase in a cap-independent way. (**D**) The ratio of Renilla and the firefly fluorescence signal was quantified in healthy control cell lines (1306 and FF95) and disease cell lines (XP56BR and XP118BR). Data are represented as mean ± SD of at least three independent experiments. ns p > 0.05, * p ≤ 0.05, ** p ≤ 0.01, *** p ≤ 0.001, **** p ≤ 0.0001

In previous works, we could show that CSA and CSB cells are characterized by an activation of the unfolded protein response (UPR) (Alupei et al. 2018; Qiang et al. 2021) as a consequence of misfolded proteins originating from error-prone protein synthesis at the ribosome. As the XPG cases did not display protein translation disturbances or signs of misfolded proteins, we wondered if we could find activated stress signaling pathways common with CSA/CSB cases. In fact, monitoring the phosphorylation status of eIF2alpha, we detected a strong phosphorylation of eIF2alpha in the severe CS patient cell line (Fig. 4A). The eukaryotic initiation factor 2 alpha is one main effector of the UPR, but also an executor of the ISR and can be phosphorylated by 4 different kinases (protein kinase R-like endoplasmic reticulum kinase (PERK), protein kinases R (PKR), general control non-derepressible-2 (GCN2), heme-regulated inhibitor (HRI)). Phosphorylation of eIF2alpha results in a strong repression of cap-dependent translation initiation and preferential translation of stress-response mRNA by internal ribosomal entry sites and a repression of RNA polymerase I transcription (DuRose et al. 2009) as evident in the severe XPG/CS case. Testing overall translation initiation, we can show that translation is significantly attenuated in the XPCS1RO cell line (Fig. 4B), in line with the elevated eIF2alpha phosphorylation. Next, we provoked endoplasmic reticulum (ER) stress by tunicamycin, a stressor that forced CSA and CSB cells to undergo apoptosis (Alupei et al. 2018). Tunicamycin did elevate the unfolded protein response apoptosis inducer C/EBP homologous protein (CHOP) in the XPCS1RO cell line (supplemental Fig. 5). However, other parameters of the UPR were not elevated (supplemental Fig. 5), arguing against a general hypersensitivity of CS cells to ER-stress. Interestingly, transfection of a luciferase-reporter that distinguishes between cap-dependent (renilla) and cap-independent, IRES-dependent translation (firefly) shows a shift towards IRES-dependent translation in the severe CS case (Fig. 4C). This suggests that there might be a stress-translatome at play in severe CS.

### Reactive oxygen species and oxidative damage in *XPG*-mutant cells

**Fig. 5:**
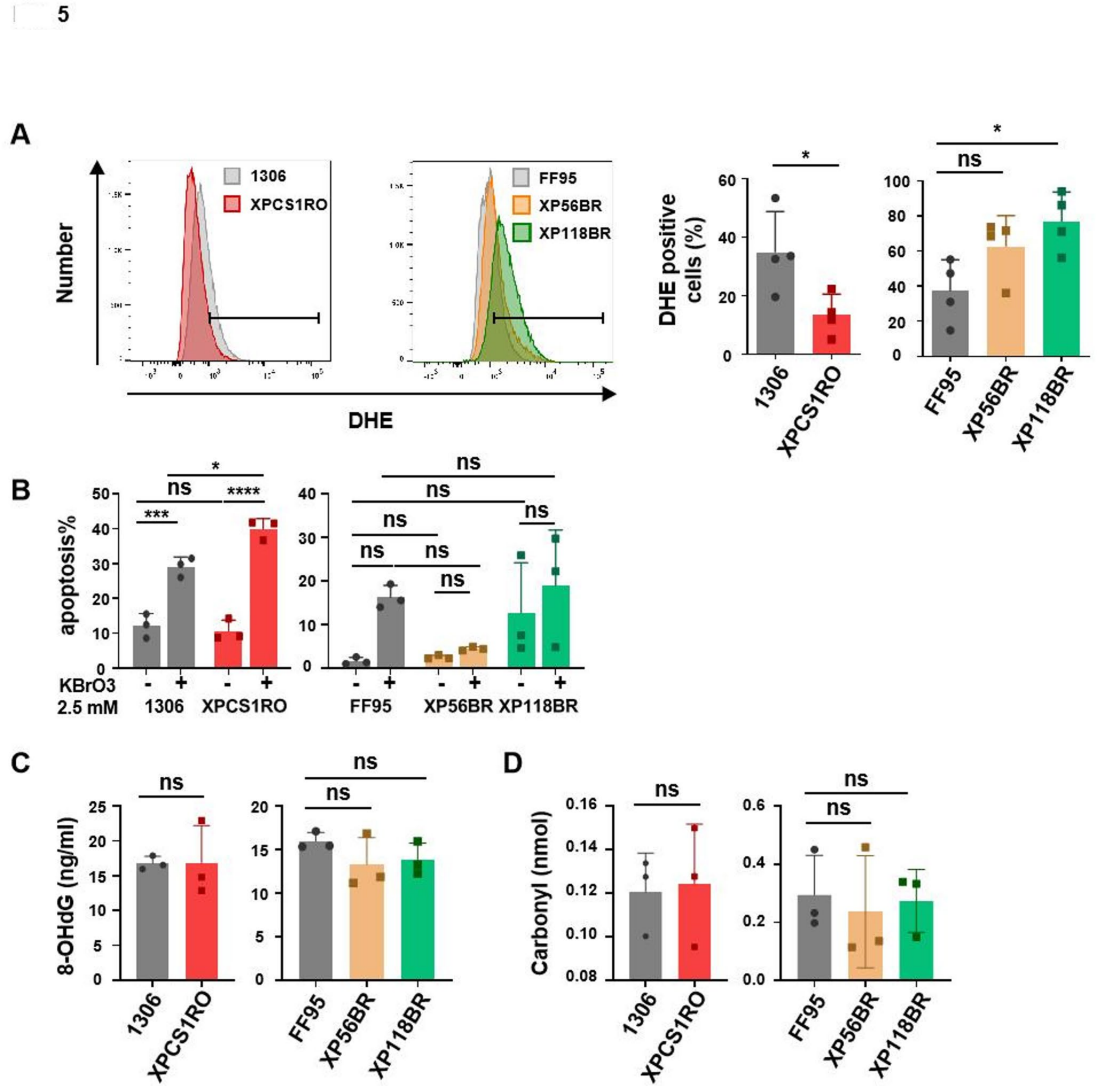
(**A**) ROS level in wild type cell lines and disease cell lines was assessed by DHE fluorescence dye. The fluorescence signal was detected by flow cytometry, and the percentage of DHE-positive cells was represented (right). (**B**) KBrO3-induced cell apoptosis was determined by PI and Annexin V-FITC double staining. The same number of cells was seeded in 6-well dishes. After being treated with KBrO3 (2.5mM) for 24 h, cells were stained with PI and Annexin V-FITC, and apoptotic cells were regarded as PI and/or Annexin V-positive cells. (**C**) DNA oxidation lesions in control cell lines and disease cell lines were assessed by detecting 8-OHdG levels via ELISA. (**D**) Protein oxidation was detected by measuring the DNPH-labelled carbonyl groups. Data are represented as mean ± SD of at least three independent experiments. ns p > 0.05, * p ≤ 0.05, ** p ≤ 0.01, *** p ≤ 0.001, **** p ≤ 0.0001

As CS cells, as well as cells from the mild UVs syndrome, suffer from elevated reactive oxygen species (ROS), presumably a consequence of mitochondrial affection (Alupei et al. 2018; Kamenisch et al. 2010; Okur et al. 2020) we investigated ROS and oxidative damage in the *XPG* mutant cells. Dihydroethidium (DHE) staining detects superoxide anions and can be quantified by flow cytometry analysis. The severe CS cell line XPCS1RO displays only faint DHE staining, indicating that mitochondrial dysfunction might not be the underlying mechanism of the phenotype, whereas the XP cell line did in fact display elevated ROS production (Fig. 5A). The CS factors are all involved in NER, which repairs helix-distorting lesions, mainly provoked by UV-light. A failure to repair UV-lesions does explain the cancer-prone disease xeroderma pigmentosum, but not childhood and neurodegeneration in CS. The mild skin disease UV-sensitive syndrome displays the same cellular inability to repair transcription-blocking lesions (TBL) like cells from CS but does not show oxidative hypersensitivity, typical for CSA or CSB-mutant CS cells. This is thought to be caused by deficient repair of oxidative DNA-lesions (Krokidis et al. 2020). Using potassium bromate to induce ROS and quantifying apoptosis by AnnexinV and PI double staining and flow cytometry, we detected an elevated apoptosis rate in the severe CS case, but not in mild CS or XP (Fig. 5B). Moreover, quantifying 8-hydroxydesoxyguanosine (8-OHdG) in genomic DNA of the CS and XP cases did not reveal enhanced DNA oxidation, neither in the severe CS nor in the XP cell line that did display elevated ROS. Our group reported enhanced protein carbonylation in CSA and CSB mutant CS cases; however, in the mutated *XPG* cell lines investigated in this study, we could not identify elevated protein carbonylation, in line with the result that we could not detect elevated error-rate of protein synthesis in the XPG/CS cases. Protein carbonylation is thought to mirror transcriptional and translational protein synthesis defects (Dukan et al. 2000). Taken together, our results implicate that the strong phosphorylation of eIF2alpha might contribute to, or cause childhood degeneration in the severe XPG/CS case.

## Discussion

XP and CS are different diseases. Whereas XP is a classical DNA-repair disorder with highly elevated cancer risk, “classical” CS, provoked by mutations in the transcription-coupled NER factors CSA or CSB, is cancer-free. As DNA-repair is the first line of defense against genomic instability and cancer, cancer-free childhood growth retardation, premature aging, and neurological degeneration cannot be the consequence of unrepaired canonical NER. This assumption is supported by the fact that transcription-coupled NER-deficiency characterizes the UV-sensitive syndrome, which can also be caused by the mutation of CSA and CSB, but is a cancer-free, mild, and non-degenerative skin disorder (Spivak 2005). Therefore, different, additional functions of the CS-factors explain growth retardation, premature aging, and neurological degeneration. The CSB protein is involved in a multitude of functions, making it tough to identify the critical function that is responsible for childhood degeneration, whereas the CSA protein is involved in fewer cellular pathways. Mutations in the XPB and XPD subunits of TFIIH can, as mutations in *XPG*, provoke a combination of XP with CS, elevated cancer risk, and childhood degeneration. The genes that can cause CS or combinations are, besides DNA-repair proteins, transcription factors; therefore, there is also strong evidence that failures in transcriptional regulation might underlie CS, premature aging, and neurodegeneration (Costanzo et al. 2024). However, a shared pathogenetic motif between all CS-genes has not been identified yet. This motif is either a non-canonical DNA repair function by NER involving all CS factors or the failure of an alternative shared function of the CS factors. Here we investigated if we find, as described for CSA, CSB, and TFIIH mutations, aberrations in ribosomal biogenesis, function, and a subsequent loss of proteostasis. In fact, we can show that an *XPG* mutation leading to severe CS is characterized by ribosomal biogenesis disturbances and aberrant translation. These findings are in line with a recent report by Taupelet et al. (2022) demonstrating that XP/CS caused by a *XPG* mutation displays disturbances in RNA polymerase I transcription and in maturation of the primary pre-rRNA transcript. In contrast to the Taupelet study, we find a reduction in ribosomal biogenesis what might be due to different cell densities at the timepoint of analysis and different methods applied. For reproducibility we had to adjust the cells to low density. We additionally identified an 18S mature rRNA deficiency, suggesting that a maturation defect reduces the backbone of the small, decoding 40S subunit, like in other forms of CS (Qiang et al. 2021) and in the genetically related disease trichothiodystrophy (TTD) (Khalid et al. 2023; Phan et al. 2021; Zhu et al. 2023). In the reported cases of TTD and CS cells, the ribosomal biogenesis disturbances were followed by an elevated ribosomal error rate and a consecutive loss of proteostasis. Here, we could not detect this chain of events in *XPG* mutant XP/CS, indicating that proteostasis collapse is not a general feature of all forms of CS. However, a strongly phosphorylated eIF2alpha that represses cap-dependent translation and favors a stress translatome was also found in the other forms of CS and in TTD, thereby indicating that different forms of CS converge on the UPR/ISR by phosphorylation of this effector. However, we are still ignorant about the trigger of the strong phosphorylation of eIF2alpha in XP/CS, because we did not observe the ER-stress indicators or misfolded proteins that provoke eIF2alpha phosphorylation in the other forms of CS and TTD. Interestingly, eIF2alpha phosphorylation is a hallmark of neurodegenerative diseases (Bond et al. 2020) and therefore might be of relevance in the pathogenesis of CS. eIF2alpha phosphorylation represses cap-dependent and overall translation initiation as well as transcription initiation of RNA polymerase I (DuRose et al. 2009) in a feedback loop when too much misfolded proteins accumulate (Fernandez et al. 2002) in the ER as an effector of the UPR. DNA-damage accumulation can be triggered by activation of GCN2 eIF2alpha phosphorylation in the ISR (Chesnokova et al. 2017), thereby linking DNA-damage with ribosomal biogenesis. NER is also involved in the repair of oxidative DNA lesions (Kumar et al. 2020), and CS cells are hypersensitive to oxidizing agents (Bohr et al. 1998). This feature distinguishes CS from UVs cells that share the hypersensitivity to UV light. Therefore, it is argued that unrepaired oxidative DNA-damage causes neurological degeneration and dwarfism (Bohr et al. 1998). We could show that CSA and CSB oxidative hypersensitivity, detected by apoptosis rate after H_2_O_2_ exposure, could be significantly reduced by pharmaceutical chaperones that prevent protein misfolding (Alupei et al. 2018). This suggests that oxidative damage to proteins contributes to the hypersensitivity of CS-cells to oxidative agents. In the current study, we used potassium-bromate to provoke oxidative stress and could indeed observe an increase in the apoptosis rate of the severely affected CS case, in accordance with earlier reports on *XPG* mutant CS cells (Soltys et al. 2013). However, no elevated ROS, nor elevated 8-oxo-dG, and no elevated protein carbonylation could be detected in the severely affected CS cells, arguing against a dominant role of oxidative stress as a driver of CS. Patient data correlate disease severity of XP/CS with the ability of the patient cells to repair oxidative DNA-damage (Soltys et al. 2013). However, we are still ignorant regarding questions of whether there are types of oxidative transcription blocking lesions (TBL) in DNA accumulating in CS, causing the pathology, and about the exact nature of these lesions. Therefore, there are still a number of unresolved questions regarding the pathogenic events that drive CS pathology. Perhaps it helps to look at interventional strategies even when the pathophysiology is only partially understood. Nicotinamide has long been known to be reduced in CS cells, and different causes have been identified- mitochondrial deficiencies (Scheibye-Knudsen et al. 2014) and DNA-damage (Okur et al. 2020). NAD + supplementation is besides caloric restriction the only treatment regimen that significantly prolongs the lifespan of *Xpg* knockout mice (Birkisdottir et al. 2022). *Xpg* knockout mice mimic the drastic human phenotype of CS with postnatal growth failure and premature death (Harada et al. 1999), a phenotype that is not recapitulated in the CSB or CSA KO mice. Therefore, either the critical CS-cause is only reflected by *Xpg*-mutations in mice, or it is not related to CS at all. However, the fact that NAD^+^ treatment delays the pathology in this mouse model indicates that metabolic derailments are part of or driving the disease development in mice. Nonetheless, the detailed in-depth experimental analyses of CS cellular aberrations comparing XP and XP/CS can help us to identify the pathogenic events that cause CS pathology.

## Supplementary Information

Below is the link to the electronic supplementary material.

Supplementary file1 (DOCX 871 kb)

## Data Availability

No datasets were generated or analysed during the current study.

## Abbreviations

ATF 4/6: Transcription factor 4/6
CDK 7: Cyclin-dependent kinase 7
CS: Cockayne syndrome
CSA/B: Cockayne syndrome protein A/B
CHOP: C/EBP homologous protein
CrPV: Cricket paralysis virus
DNPH: 2,4-Dinitrophenyl-hydrazine
DHE: Dihydroethidium
eIF2α: Eukaryotic initiation factor 2α
ER: Endoplasmic reticulum
*ERCC*: Excision-repair cross-complementation
GCN2: General control non-derepressible-2
gDNA: Genomic DNA
HRI: Heme-regulated inhibitor
IRES: Internal ribosomal entry site
ISR: Integrated stress response
NER: Nucleotide excision repair
NLS: Nuclear localization signal
PERK: Protein kinase R-like endoplasmic reticulum kinase
PCV: Packed cell volume
PI: Propidium iodide
PKR: Protein kinases R
PS: Penicillin-treptomycin
rDNA: Ribosomal DNA
rRNA: Ribosomal RNA
ROS: Reactive oxygen species
TBL: Transcription blocking lesions
TFIIH: Transcription factor II H
TTD: Trichothiodystrophy
UV: Ultraviolet
UPR: Unfolded protein response
UBF: Upstream binding factor
XP: Xeroderma pigmentosum
XPA-G: Xeroderma pigmentosum complementation group A-G
XPV: Xeroderma pigmentosum variant

## References

[CR1] Alupei MC, Maity P, Esser PR, Krikki I, Tuorto F, Parlato R, Penzo M, Schelling A, Laugel V, Montanaro L, Scharffetter-Kochanek K, Iben S (2018) Loss of proteostasis is a pathomechanism in Cockayne syndrome. Cell Rep 23:1612–1619. 10.1016/j.celrep.2018.04.041 29742419 10.1016/j.celrep.2018.04.041

[CR2] Assfalg R, Lebedev A, Gonzalez OG, Schelling A, Koch S, Iben S (2012) TFIIH is an elongation factor of RNA polymerase I. Nucleic Acids Res 40:650–659. 10.1093/nar/gkr746 21965540 10.1093/nar/gkr746 PMC3258137

[CR3] Birkisdottir MB, van Galen I, Brandt RMC, Barnhoorn S, van Vliet N, van Dijk C, Nagarajah B, Imholz S, van Oostrom CT, Reiling E, Gyenis A, Mastroberardino PG, Jaarsma D, van Steeg H, Hoeijmakers JHJ, Dolle MET, Vermeij WP (2022) The use of progeroid DNA repair-deficient mice for assessing anti-aging compounds, illustrating the benefits of nicotinamide riboside. Front Aging 3:1005322. 10.3389/fragi.2022.1005322 36313181 10.3389/fragi.2022.1005322 PMC9596940

[CR4] Bohr V, Anson RM, Mazur S, Dianov G (1998) Oxidative DNA damage processing and changes with aging. Toxicol Lett 102:47–52. 10.1016/s0378-4274(98)00280-x 10022231 10.1016/s0378-4274(98)00280-x

[CR5] Bond S, Lopez-Lloreda C, Gannon PJ, Akay-Espinoza C, Jordan-Sciutto KL (2020) The integrated stress response and phosphorylated eukaryotic initiation factor 2alpha in neurodegeneration. J Neuropathol Exp Neurol 79:123–143. 10.1093/jnen/nlz129 31913484 10.1093/jnen/nlz129 PMC6970450

[CR6] Bradsher J, Auriol J, Proietti de Santis L, Iben S, Vonesch JL, Grummt I, Egly JM (2002) CSB is a component of RNA pol I transcription. Mol Cell 10:819–829. 10.1016/s1097-2765(02)00678-0 12419226 10.1016/s1097-2765(02)00678-0

[CR7] Brooks PJ (2013) Blinded by the UV light: how the focus on transcription-coupled NER has distracted from understanding the mechanisms of Cockayne syndrome neurologic disease. DNA Repair (Amst) 12:656–671. 10.1016/j.dnarep.2013.04.018 23683874 10.1016/j.dnarep.2013.04.018 PMC4240003

[CR8] Bukowska B, Karwowski BT (2018) Actual state of knowledge in the field of diseases related with defective nucleotide excision repair. Life Sci 195:6–18. 10.1016/j.lfs.2017.12.035 29305302 10.1016/j.lfs.2017.12.035

[CR9] Chesnokova E, Bal N, Kolosov P (2017) Kinases of eIF2a Switch Translation of mRNA Subset during Neuronal Plasticity. Int J Mol Sci 18. 10.3390/ijms18102213 10.3390/ijms18102213 PMC5666893 29065505

[CR10] Costanzo F, Paccosi E, Proietti-De-Santis L, Egly JM (2024) CS proteins and ubiquitination: orchestrating DNA repair with transcription and cell division. Trends Cell Biol 34:882–895. 10.1016/j.tcb.2024.06.002 38910038 10.1016/j.tcb.2024.06.002

[CR11] Dukan S, Farewell A, Ballesteros M, Taddei F, Radman M, Nystrom T (2000) Protein oxidation in response to increased transcriptional or translational errors. Proc Natl Acad Sci USA 97:5746–5749. 10.1073/pnas.100422497 10811907 10.1073/pnas.100422497 PMC18504

[CR12] DuRose JB, Scheuner D, Kaufman RJ, Rothblum LI, Niwa M (2009) Phosphorylation of eukaryotic translation initiation factor 2alpha coordinates rRNA transcription and translation inhibition during endoplasmic reticulum stress. Mol Cell Biol 29:4295–4307. 10.1128/MCB.00260-09 19470760 10.1128/MCB.00260-09 PMC2715810

[CR13] Egly JM (2001) The 14th Datta lecture. TFIIH: from transcription to clinic. FEBS Lett 498:124–128. 10.1016/s0014-5793(01)02458-9 11412842 10.1016/s0014-5793(01)02458-9

[CR14] Emmert S, Schneider TD, Khan SG, Kraemer KH (2001) The human XPG gene: gene architecture, alternative splicing and single nucleotide polymorphisms. Nucleic Acids Res 29:1443–1452. 10.1093/nar/29.7.1443 11266544 10.1093/nar/29.7.1443 PMC31292

[CR15] Fassihi H, Sethi M, Fawcett H, Wing J, Chandler N, Mohammed S, Craythorne E, Morley AM, Lim R, Turner S, Henshaw T, Garrood I, Giunti P, Hedderly T, Abiona A, Naik H, Harrop G, McGibbon D, Jaspers NG, Botta E, Nardo T, Stefanini M, Young AR, Sarkany RP, Lehmann AR (2016) Deep phenotyping of 89 xeroderma pigmentosum patients reveals unexpected heterogeneity dependent on the precise molecular defect. Proc Natl Acad Sci USA 113:E1236–E1245. 10.1073/pnas.1519444113 26884178 10.1073/pnas.1519444113 PMC4780618

[CR16] Fernandez J, Yaman I, Sarnow P, Snider MD, Hatzoglou M (2002) Regulation of internal ribosomal entry site-mediated translation by phosphorylation of the translation initiation factor eIF2alpha. J Biol Chem 277:19198–19205. 10.1074/jbc.M201052200 11877448 10.1074/jbc.M201052200

[CR17] Ferri D, Orioli D, Botta E (2020) Heterogeneity and overlaps in nucleotide excision repair disorders. Clin Genet 97:12–24. 10.1111/cge.13545 30919937 10.1111/cge.13545

[CR18] Hamel BC, Raams A, Schuitema-Dijkstra AR, Simons P, van der Burgt I, Jaspers NG, Kleijer WJ (1996) Xeroderma pigmentosum–Cockayne syndrome complex: a further case. J Med Genet 33:607–610. 10.1136/jmg.33.7.607 8818951 10.1136/jmg.33.7.607 PMC1050673

[CR19] Harada YN, Shiomi N, Koike M, Ikawa M, Okabe M, Hirota S, Kitamura Y, Kitagawa M, Matsunaga T, Nikaido O, Shiomi T (1999) Postnatal growth failure, short life span, and early onset of cellular senescence and subsequent immortalization in mice lacking the xeroderma pigmentosum group G gene. Mol Cell Biol 19:2366–2372. 10.1128/MCB.19.3.2366 10022922 10.1128/mcb.19.3.2366 PMC84028

[CR20] Hartmann M, Neher L, Grupp B, Cao Z, Chiew C, Iben S (2025) Development of a highly sensitive method to detect translational infidelity. Biol Methods Protoc 10:bpaf008. 10.1093/biomethods/bpaf008 39925782 10.1093/biomethods/bpaf008 PMC11805343

[CR21] Ito S, Kuraoka I, Chymkowitch P, Compe E, Takedachi A, Ishigami C, Coin F, Egly JM, Tanaka K (2007) XPG stabilizes TFIIH, allowing transactivation of nuclear receptors: implications for Cockayne syndrome in XP-G/CS patients. Mol Cell 26:231–243. 10.1016/j.molcel.2007.03.013 17466625 10.1016/j.molcel.2007.03.013

[CR22] Iyer N, Reagan MS, Wu KJ, Canagarajah B, Friedberg EC (1996) Interactions involving the human RNA polymerase II transcription/nucleotide excision repair complex TFIIH, the nucleotide excision repair protein XPG, and Cockayne syndrome group B (CSB) protein. Biochemistry 35:2157–2167. 10.1021/bi9524124 8652557 10.1021/bi9524124

[CR23] Kamenisch Y, Fousteri M, Knoch J, von Thaler AK, Fehrenbacher B, Kato H, Becker T, Dolle ME, Kuiper R, Majora M, Schaller M, van der Horst GT, van Steeg H, Rocken M, Rapaport D, Krutmann J, Mullenders LH, Berneburg M (2010) Proteins of nucleotide and base excision repair pathways interact in mitochondria to protect from loss of subcutaneous fat, a hallmark of aging. J Exp Med 207:379–390. 10.1084/jem.20091834 20100872 10.1084/jem.20091834 PMC2822596

[CR24] Khalid F, Phan T, Qiang M, Maity P, Lasser T, Wiese S, Penzo M, Alupei M, Orioli D, Scharffetter-Kochanek K, Iben S (2023) TFIIH mutations can impact on translational fidelity of the ribosome. Hum Mol Genet 32:1102–1113. 10.1093/hmg/ddac268 36308430 10.1093/hmg/ddac268 PMC10026254

[CR25] Koch S, Garcia Gonzalez O, Assfalg R, Schelling A, Schafer P, Scharffetter-Kochanek K, Iben S (2014) Cockayne syndrome protein A is a transcription factor of RNA polymerase I and stimulates ribosomal biogenesis and growth. Cell Cycle 13:2029–2037. 10.4161/cc.29018 24781187 10.4161/cc.29018 PMC4111694

[CR26] Kraemer KH, DiGiovanna JJ, Tamura D (1993) Xeroderma Pigmentosum. In: Adam MP, Feldman J, Mirzaa GM, Pagon RA, Wallace SE, Amemiya A (eds) GeneReviews((R)), Seattle (WA) 20301571

[CR27] Kraemer KH, Lee MM, Scotto J (1987) Xeroderma pigmentosum. Cutaneous, ocular, and neurologic abnormalities in 830 published cases. Arch Dermatol 123:241–250. 10.1001/archderm.123.2.241 3545087 10.1001/archderm.123.2.241

[CR28] Krokidis MG, D'Errico M, Pascucci B, Parlanti E, Masi A, Ferreri C, Chatgilialoglu C (2020) Oxygen-dependent accumulation of purine DNA lesions in cockayne syndrome cells. Cells 9. 10.3390/cells9071671 10.3390/cells9071671 PMC7407219 32664519

[CR29] Kumar N, Raja S, Van Houten B (2020) The involvement of nucleotide excision repair proteins in the removal of oxidative DNA damage. Nucleic Acids Res 48:11227–11243. 10.1093/nar/gkaa777 33010169 10.1093/nar/gkaa777 PMC7672477

[CR30] Kuper J, Kisker C (2023) At the core of nucleotide excision repair. Curr Opin Struct Biol 80:102605. 10.1016/j.sbi.2023.102605 37150041 10.1016/j.sbi.2023.102605

[CR31] Lopez-Otin C, Blasco MA, Partridge L, Serrano M, Kroemer G (2013) The hallmarks of aging. Cell 153:1194–1217. 10.1016/j.cell.2013.05.039 23746838 10.1016/j.cell.2013.05.039 PMC3836174

[CR32] Lopez-Otin C, Blasco MA, Partridge L, Serrano M, Kroemer G (2023) Hallmarks of aging: an expanding universe. Cell 186:243–278. 10.1016/j.cell.2022.11.001 36599349 10.1016/j.cell.2022.11.001

[CR33] Nouspikel T, Lalle P, Leadon SA, Cooper PK, Clarkson SG (1997) A common mutational pattern in Cockayne syndrome patients from xeroderma pigmentosum group G: implications for a second XPG function. Proc Natl Acad Sci U S A 94:3116–3121. 10.1073/pnas.94.7.3116 9096355 10.1073/pnas.94.7.3116 PMC20331

[CR34] Okur MN, Fang EF, Fivenson EM, Tiwari V, Croteau DL, Bohr VA (2020) Cockayne syndrome proteins CSA and CSB maintain mitochondrial homeostasis through NAD(+) signaling. Aging Cell 19:e13268. 10.1111/acel.13268 33166073 10.1111/acel.13268 PMC7744955

[CR35] Pal R, Paul N, Bhattacharya D, Rakshit S, Shanmugam G, Sarkar K (2022) XPG in the nucleotide excision repair and beyond: a study on the different functional aspects of XPG and its associated diseases. Mol Biol Rep 49:7995–8006. 10.1007/s11033-022-07324-1 35596054 10.1007/s11033-022-07324-1

[CR36] Penzo M, Carnicelli D, Montanaro L, Brigotti M (2016) A reconstituted cell-free assay for the evaluation of the intrinsic activity of purified human ribosomes. Nat Protoc 11:1309–1325. 10.1038/nprot.2016.072 27336708 10.1038/nprot.2016.072

[CR37] Phan T, Maity P, Ludwig C, Streit L, Michaelis J, Tsesmelis M, Scharffetter-Kochanek K, Iben S (2021) Nucleolar TFIIE plays a role in ribosomal biogenesis and performance. Nucleic Acids Res 49:11197–11210. 10.1093/nar/gkab866 34581812 10.1093/nar/gkab866 PMC8565312

[CR38] Qiang M, Khalid F, Phan T, Ludwig C, Scharffetter-Kochanek K, Iben S (2021) Cockayne syndrome-associated CSA and CSB mutations impair ribosome biogenesis, ribosomal protein stability, and global protein folding. Cells 10. 10.3390/cells10071616 10.3390/cells10071616 PMC8306422 34203326

[CR39] Scheibye-Knudsen M, Mitchell SJ, Fang EF, Iyama T, Ward T, Wang J, Dunn CA, Singh N, Veith S, Hasan-Olive MM, Mangerich A, Wilson MA, Mattson MP, Bergersen LH, Cogger VC, Warren A, Le Couteur DG, Moaddel R, Wilson DM, Croteau DL, de Cabo R, Bohr VA (2014) A high-fat diet and NAD(+) activate Sirt1 to rescue premature aging in cockayne syndrome. Cell Metab 20:840–855. 10.1016/j.cmet.2014.10.005 25440059 10.1016/j.cmet.2014.10.005 PMC4261735

[CR40] Soltys DT, Rocha CR, Lerner LK, de Souza TA, Munford V, Cabral F, Nardo T, Stefanini M, Sarasin A, Cabral-Neto JB, Menck CF (2013) Novel XPG (ERCC5) mutations affect DNA repair and cell survival after ultraviolet but not oxidative stress. Hum Mutat 34:481–489. 10.1002/humu.22259 23255472 10.1002/humu.22259

[CR41] Spivak G (2005) Uv-sensitive syndrome. Mutat Res 577:162–169. 10.1016/j.mrfmmm.2005.03.017 15916784 10.1016/j.mrfmmm.2005.03.017

[CR42] Taupelet F, Donnio LM, Magnani C, Mari PO, Giglia-Mari G (2022) A stable XPG protein is required for proper ribosome biogenesis: insights on the phenotype of combinate Xeroderma Pigmentosum/Cockayne Syndrome patients. PLoS ONE 17:e0271246. 10.1371/journal.pone.0271246 35802638 10.1371/journal.pone.0271246 PMC9269744

[CR43] Treaster SB, Ridgway ID, Richardson CA, Gaspar MB, Chaudhuri AR, Austad SN (2014) Superior proteome stability in the longest lived animal. Age 36:9597. 10.1007/s11357-013-9597-9 24254744 10.1007/s11357-013-9597-9 PMC4082568

[CR44] Zhu G, Khalid F, Zhang D, Cao Z, Maity P, Kestler HA, Orioli D, Scharffetter-Kochanek K, Iben S (2023) Ribosomal dysfunction is a common pathomechanism in different forms of Trichothiodystrophy. Cells. 10.3390/cells12141877 37508541 10.3390/cells12141877 PMC10377840

