# Supplementary material for: Cockayne syndrome mutation in XPG activate the integrated stress response
Source: Hum Genet. 2026 Jan 21;145(1):15. doi: 10.1007/s00439-025-02804-3 (PMC12823737; doi:10.1007/s00439-025-02804-3)
Supplement: Supplementary file 1 — Supplementary file1 (DOCX 871 kb) [file 439_2025_2804_MOESM1_ESM.docx]

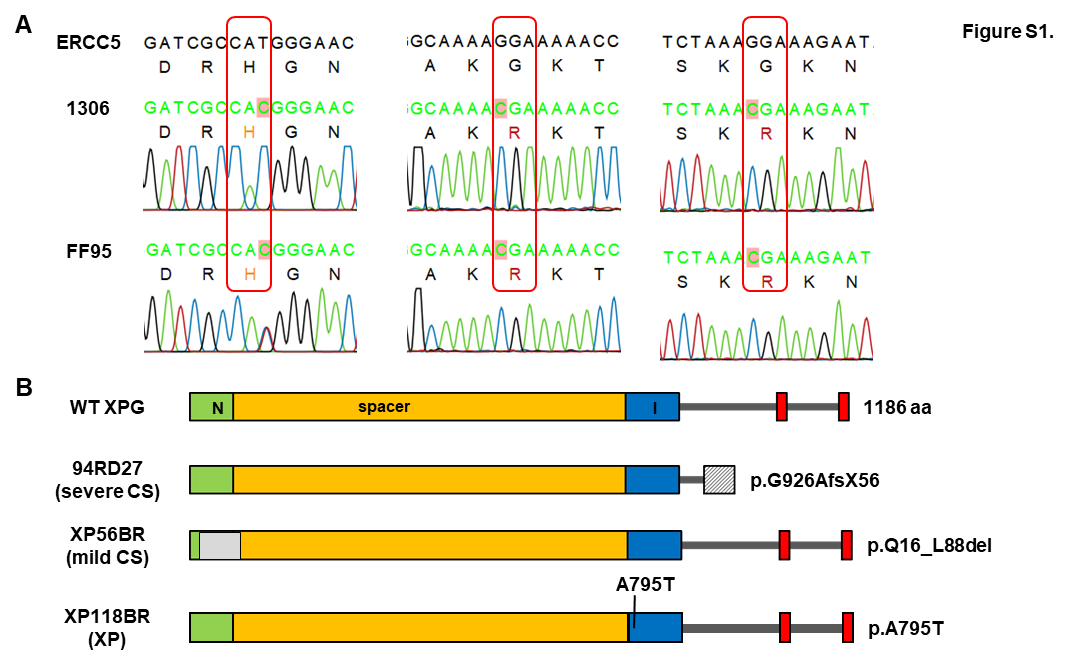


**Supplementary Figure 1.** Sequence analysis of wild type cell lines and predicted XPG protein structure in all cell lines used in this study. (A) Sequence results of wild type cell lines (1306 and FF95) compared with *ERCC 5* gene transcript (NM_000123.4). Both 1306 and FF95 show three same mutations (c.373T>C [silent mutation], c.3392G>C [p.G1053R], and c.3473G>C [p.G1080R]). (B) Schematic diagram of wild type XPG protein and predicted XPG proteins in CS and/or XP patients. Green and blue areas represent the N-terminal domain and internal domain (I-domain) in XPG protein, respectively. Red frames stand for nuclear localization signals (NLS). The rectangle with slashes in the XPCS1RO cell line represents 56 amino acids that are not related to XPG after a missense mutation. The gray rectangle in XP56BR means a deletion of 16-88 aa in the XPG protein. Predicted XPG proteins in XPCS1RO and XP118BR were based on our sequence results, while XP56BR was based on the reference of Fassihi et al ([Fassihi et al. 2016](#_ENREF_15)).


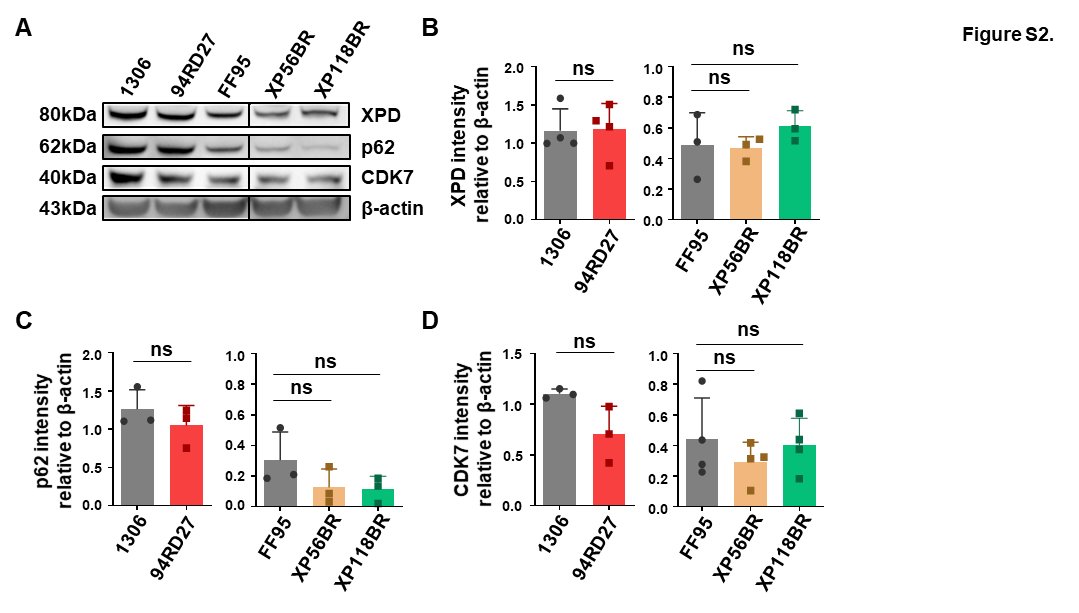


**Supplementary Figure 2.** (A) Western blot analysis of TFIIH subunits (XPD, p62, and cyclin-dependent kinase 7 (CDK7) ) expression in wild type controls and disease cell lines. (B)-(D) Abundance of TFIIH subunits and the values were quantified and normalized with β-actin. Data are represented as mean ± SD of at least three independent experiments. ns p > 0.05, * p ≤ 0.05, ** p ≤ 0.01, *** p ≤ 0.001, **** p≤ 0.0001.

**
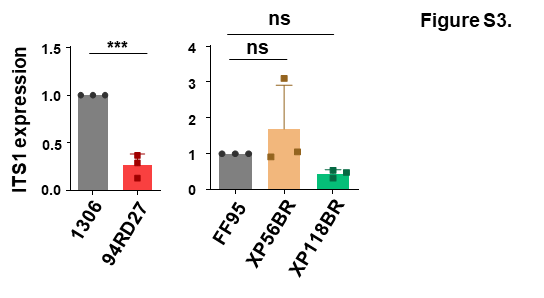
**

**Supplementary Figure 3.** qPCR analysis of ITS 1 expression in wild type controls and *XPG* mutated cell lines. The values were normalized with β-actin. Data are represented as mean ± SD of at least three independent experiments. ns p > 0.05, * p ≤ 0.05, ** p ≤ 0.01, *** p ≤ 0.001, **** p≤ 0.0001.

**
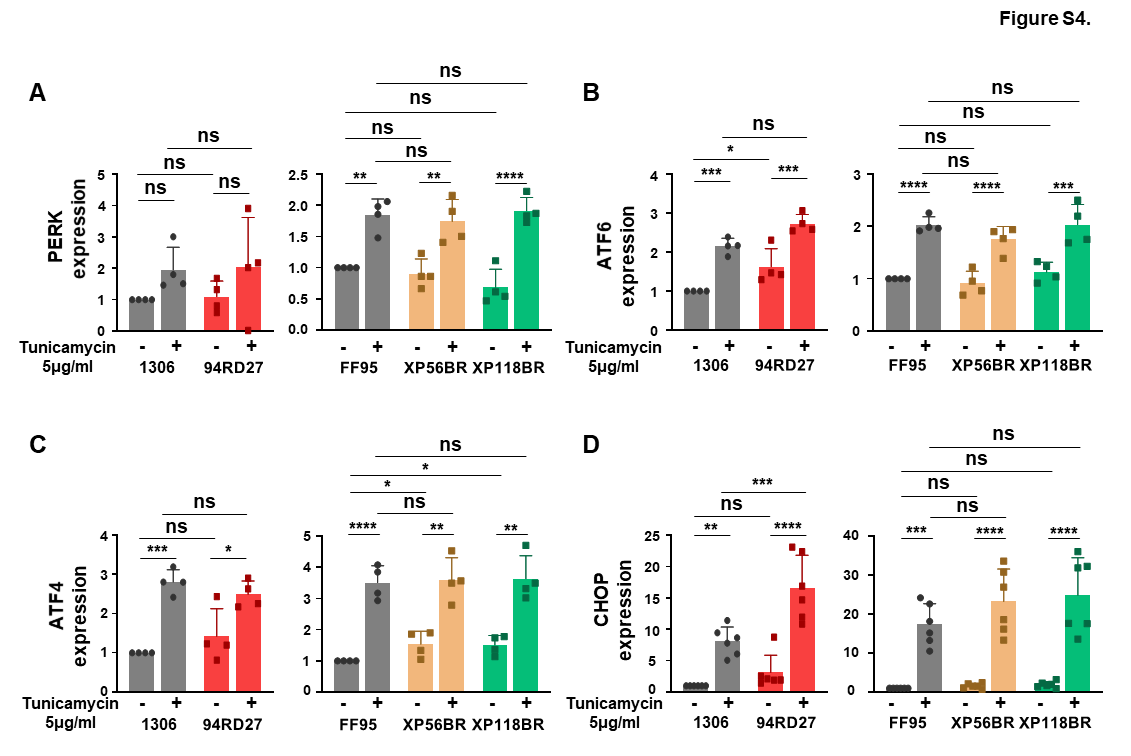
**

**Supplementary Figure 4.** (A)-(D) ER stress-related gene expression (PERK, transcription factor (ATF6), transcription factor 4 (ATF4), and CHOP) detected via qPCR. Cells (wild type cell lines and disease cell lines) were treated with tunicamycin (5μg/ml) for 6 h to induce ER stress. The same volume of DMSO was added as a negative control. CT values were normalized with β-actin. Data are represented as mean ± SD of at least three independent experiments. ns p > 0.05, * p ≤ 0.05, ** p ≤ 0.01, *** p ≤ 0.001, **** p≤ 0.0001.

**
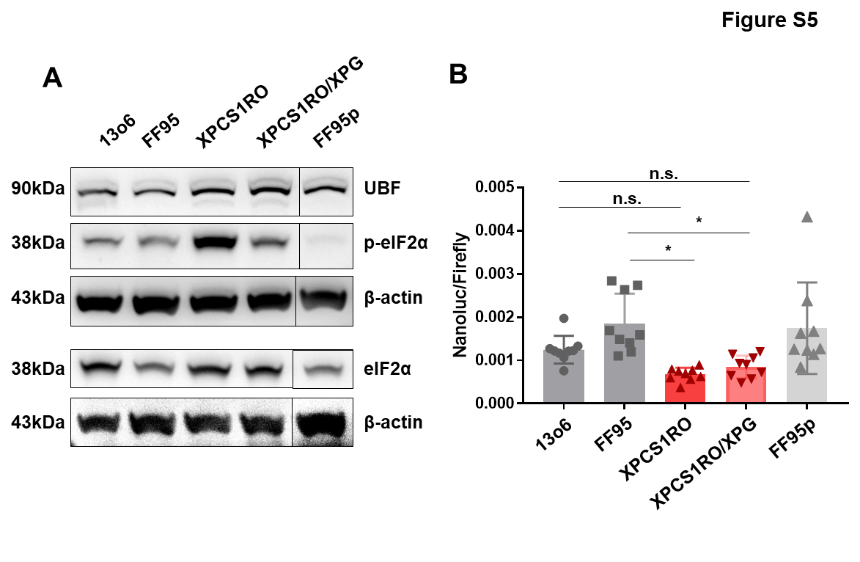
**

**Supplementary Figure 5.** (A) Western blot analysis of UBF, phosphorylated eIF2alpha (p-eIF2α), and total eIF2alpha (eIF2α) in wild type control cell lines (1306 and FF95), XPG-deficient cell line (XPCS1RO), XPG reconstituted cells, and a primary fibroblast line (FF95p). β-actin was used as a loading control. (B) Translational fidelity assay conducted in 1306, FF95, XPCS1RO, XPG reconstituted cells, and FF95p. Cells were co-transfected with a mutated Nano luciferase reporter and a firefly luciferase control reporter. For each cell line, transfection was performed in three independent replicates on the same day, and each replicate was plated into three wells (n = 9 total). Data are represented as mean ± SD. ns p > 0.05, * p ≤ 0.05, ** p ≤ 0.01, *** p ≤ 0.001, **** p ≤ 0.0001.

**
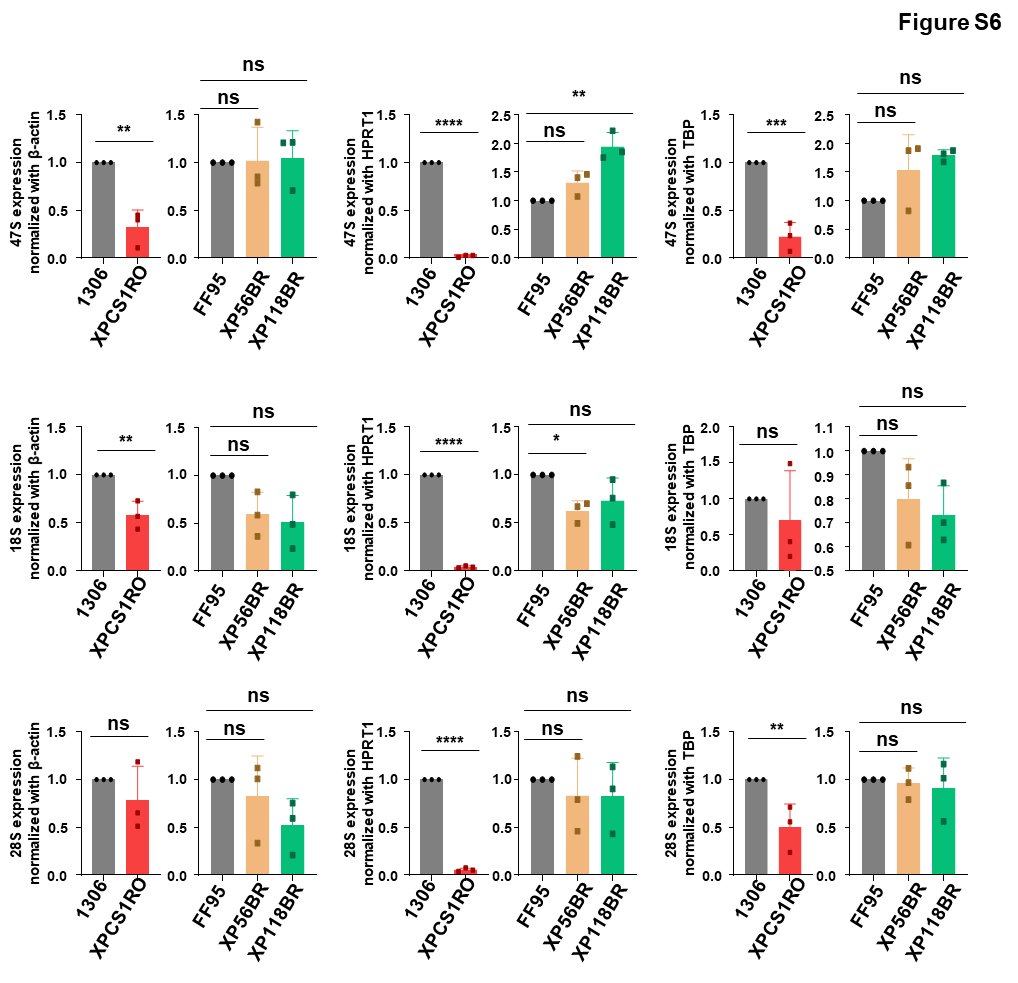
**

**
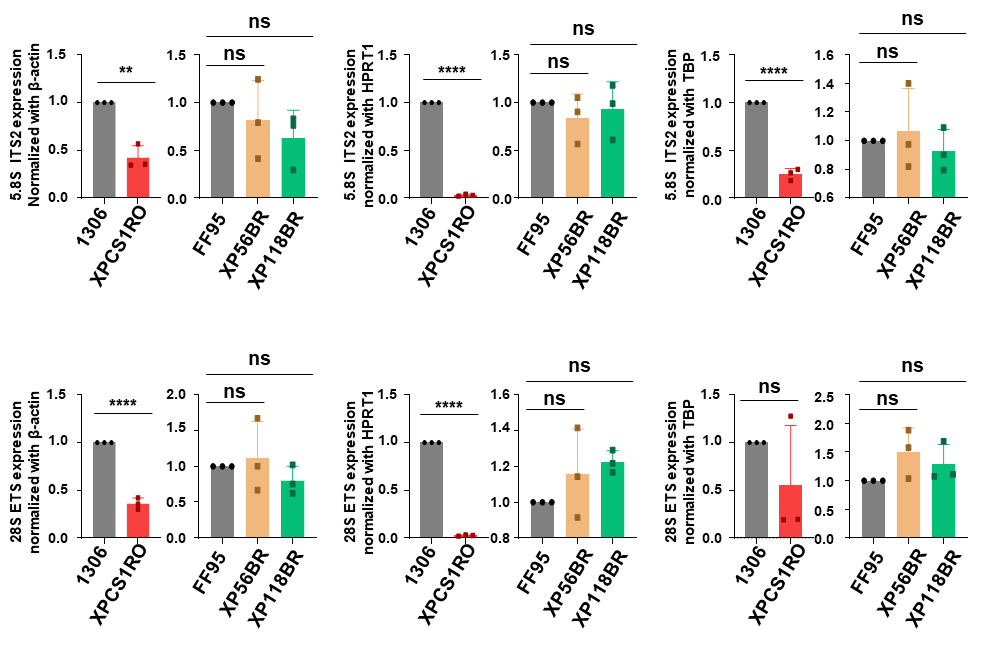
**

**Supplementary Figure 6.** qRT-PCR analysis of rRNA including 47S, 18S, 28S, 5.8S-ITS2, and 28S-ETS in wild type controls and XPG-mutated cell lines. The experiment was performed to validate the results shown in the main figure using different housekeeping genes (β-actin, HPRT1, and TBP) for normalization. Data are represented as mean ± SD of at least three independent experiments. ns p > 0.05, * p ≤ 0.05, ** p ≤ 0.01, *** p ≤ 0.001, **** p ≤ 0.0001.

**Supplementary tables:**

**Supplementary Table 1** Cell lines information used in this study

| **Cell lines** | **Immortalization** | **Phenotypes** | **Mutated nucleotide** | **Predicted proteins** | **Exon** | Clinical features | **References** |
| --- | --- | --- | --- | --- | --- | --- | --- |
| 1306 | SV40 transformed cell line | wild type | - | - | - | Healthy control | Department of Dermatology |
| FF95 | hTert transformed cell line | wild type | - | - | - | Healthy control | Department of Dermatology |
| XPCS1RO hom. | SV40 transformed patient cell line | XP/CS | c.2972delT | p.G926AfsX56 | 13 | Severe early-onset CS, died at 7 months | (Hamel et al. 1996; Nouspikel et al. 1997) |
| XPCS1RO / XPG | SV40 transformed patient cell line | XP/CS | - | - | - | XPG reconstituted cell line | (Taupelet et al. 2022) |
| FF95p | Primary cell line | wild type | - | - | - | Healthy control | Department of Dermatology |
| XP56BR hom. | hTert transformed patient cell line | XP/CS | c.264+1delG | p.Q16_L88del | 1-2 | mild CS | (Fassihi et al. 2016) |
| XP118BR het. | hTert transformed patient cell line | XP | c.2383G>A c.1842delT | p.A795T p.L615WfsX17 | 11; 8 | XP without neurodegeneration | (Fassihi et al. 2016) |
| **Abbreviations: hom., homozygous; het., heterozygous; XP, xeroderma pigmentosum. CS, Cockayne syndrome.** | | | | | | | |

**Supplementary Table 2** Primers used in cDNA products in ERCC 5 gene sequencing

| **primers** | | **Sequence 5’-3’** |
| --- | --- | --- |
| 1 | Forward | ATTTAGGTGACACTATAGAACTCTTAGGACGCAGCCGCC |
|  | Reverse | AATTAACCCTCACTAAAGGGCTTTTGGACATGTTCTATATGCTGGTTC |
| 2 | Forward | GTAAAACGACGGCCAGTGAAGCAATGCCAGAGGAGTC |
|  | Reverse | CCGTAACTTGAAAGTATTTCGTTAGTGCTGGCTACGTGCTCC |
| 3 | Forward | GTAAAACGACGGCCAGGTGATGAAGGACTTAAAGTGAGAGATGG |
|  | Reverse | CAGGAAACAGCTATGACGCTTGAAGTTCCTCATCACTAATCACAC |
| 4 | Forward | TAATACGACTCACTATAGGCAAAGGCCGTGGAACCAATG |
|  | Reverse | CAGGAAACAGCTATGACTTCGGTATAATCACTTCCAAGC |
| 5 | Forward | CAGGAAACAGCTATGACTGGAGCGCGGCATGTCTATAG |
|  | Reverse | TAATACGACTCACTATAGGACTGCTTCTATTTCGCTGGCTG |
| 6 | Forward | CAGGAAACAGCTATGACGATGCTAAACGTATTAAGAGCCAGAG |
|  | Reverse | TAATACGACTCACTATAGGATGGCTGTCATAACTAATTATAGAGGATAC |

**Supplementary Table 3** Primers used in gDNA products in ERCC 5 gene sequencing

| **Primer** | **Forward 5’-3’** | **Reverse 5’-3’** |
| --- | --- | --- |
| Exon and intron 2 | GGATCGCCATGGGAACTCAA | TCATTGTACCCATGATGAACTCTC |
| Exon and intron 8 | GCAGCAGAACGAACTTTGCC | CATGACCAGCTGTGACTCCC |

**Supplementary Table 4** Primers used in RT-qPCR

| **Primer** | **Forward 5’-3’** | **Reverse 5’-3’** |
| --- | --- | --- |
| XPG 1 | CAAGCACTTAAAGGAGTCCGGGAT | TTTCCTGGAGTCACTGGACGCTAA |
| XPG 2 | AAAGGAGAACAGCTGCGAAAGAGC | CCTCGCACGTCTTAGTTTCCTTCT |
| 47S | TGTCAGGCGTTCTCGTCTC | AGCACGACGTCACCACATC |
| 5.8S/ITS2 | TCGTGCGTCGATGAAGAACGCAG | ATTGATCGGCAAGCGACGCTCAG |
| 28S/ETS | GGTTTCGTACGTAGCAGAGCAGC | AGGGAGGAAGACGAACGGAAGGAC |
| 18S | CGATGCGGCGGCGTTATTCC | GAACGGCCATGCACCACCAC |
| 28S | AGTCGGGTTGCTTGGGAATGC | CCCTTACGGTACTTGTTGACT |
| ITS1 | GCGGAGGTTTAAAGACCCCTTGG | GTCGGAAGGTTTCACACCACGG |
| CHOP | GCACCTCCCAGAGCCCTCACTCTCC | GTCTACTCCAAGCCTTCCCCCTGCG |
| ATF 4 | TTAAGCCATGGCGCTTCTCA | CATTTCGGTCATGTTGCGGT |
| ATF 6 | TTTTAGCCCGGGACTCTTTC | TCAGCAAAGAGAGCAGAATCC |
| PERK | ATCCCCCATGGAACGACCTG | ACCCGCCAGGGACAAAAATG |
| β-actin | AATGTCGCGGAGGACCTTTGATTGC | AGGATGGCAAGGGACTTCCTGTAA |

**Supplementary Table 5** Antibodies used in Western blot analysis

| **Primary antibody** | **Source** |
| --- | --- |
| Mouse monoclonal antibody against p62 | Santa Cruz Biotechnology, sc-25329 |
| Mouse monoclonal antibody against XPD | Abcam, Ab54676 |
| Mouse monoclonal antibody against cdk7 | Santa Cruz Biotechnology, sc-7344 (C-4) |
| Rabbit polyclonal antibody against phosphorylated eIF2α (Ser51) | Cell signaling, 9721s |
| Rabbit polyclonal antibody against eIF2α | Cell signaling, 9722s |
| Mouse monoclonal antibody against UBF | Santa Cruz Biotechnology, sc-13125 |
| **Secondary antibody** | **Source** |
| Mouse monoclonal β-actin (C4) horseradish peroxidase (HRP) | Sanat Cruz Biotechnology, sc-47778 |
| Goat anti-rabbit IgG (H&L), HRP conjugate | ImmunoReagents Inc, GtxRb-003-DHRPX |
| Goat anti-mouse IgG (H&L), HRP conjugate | ImmunoReagents Inc, GtxMu-003-DHRPX |

**Supplementary Table 6** Materials used in in vitro translation assay

| **Component** | **Volume** |
| --- | --- |
| CrPV-IRES RNA | 600ng |
| 10×TM | 4μl |
| Cell extract | 40-70μg protein |
| Nuclease-free water | Fill up to 40μl |
| Total | 40μl |

**Supplementary Table 7-1** Raw data of *in vitro* translation assay (Figure 4D) of the cell lines 1306 and XPCS1RO

| **Repetition** | **Cell lines** | **Firefly** | **Renilla** | **R/F** | **Average** |
| --- | --- | --- | --- | --- | --- |
| 1 | 1306 | 228300 | 2070000 | 9,07 | 8,81 |
|  |  | 235800 | 2019000 | 8,56 |  |
|  | XPCS1RO | 102500 | 648400 | 6,33 | 6,16 |
|  |  | 112000 | 672100 | 6,00 |  |
| 2 | 1306 | 273400 | 1821000 | 6,66 | 6,53 |
|  |  | 283400 | 1811000 | 6,39 |  |
|  | XPCS1RO | 200800 | 790100 | 3,93 | 5,04 |
|  |  | 61620 | 378500 | 6,14 |  |
| 3 | 1306 | 174700 | 1275000 | 7,30 | 7,15 |
|  |  | 140500 | 984400 | 7,01 |  |
|  | XPCS1RO | 70960 | 569600 | 8,03 | 6,20 |
|  |  | 185000 | 808400 | 4,37 |  |
| 4 | 1306 | 110400 | 705200 | 6,39 | 6,02 |
|  |  | 114300 | 647000 | 5,66 |  |
|  | XPCS1RO | 82880 | 359600 | 4,34 | 4,45 |
|  |  | 82020 | 374300 | 4,56 |  |
| 5 | 1306 | 119400 | 818200 | 6,85 | 6,62 |
|  |  | 105600 | 675000 | 6,39 |  |
|  | XPCS1RO | 140600 | 701000 | 4,99 | 5,70 |
|  |  | 53920 | 346200 | 6,42 |  |

**Supplemental Table 7-2** Raw data of *in vitro* translation assay (Figure 4D) of the cell lines FF95, XP56BR and XP118BR

| **Repetition** | **Cell line** | **Firefly** | **Renilla** | **R/F** | **Average** |
| --- | --- | --- | --- | --- | --- |
| 1 | FF95 | 974800 | 1073000 | 1,10 | 1,31 |
|  |  | 1002000 | 1521000 | 1,52 |  |
|  | XP56BR | 569500 | 869600 | 1,53 | 1,34 |
|  |  | 452500 | 517400 | 1,14 |  |
|  | XP118BR | 289200 | 518500 | 1,79 | 1,58 |
|  |  | 244800 | 336800 | 1,38 |  |
| 2 | FF95 | 430200 | 223500 | 0,52 | 0,47 |
|  |  | 598600 | 255800 | 0,43 |  |
|  | XP56BR | 236300 | 259100 | 1,10 | 1,12 |
|  |  | 605700 | 696900 | 1,15 |  |
|  | XP118BR | 519700 | 316600 | 0,61 | 0,66 |
|  |  | 295500 | 207300 | 0,70 |  |
| 3 | FF95 | 304000 | 529800 | 1,74 | 1,85 |
|  |  | 165600 | 325000 | 1,96 |  |
|  | XP56BR | 388800 | 342700 | 0,88 | 0,90 |
|  |  | 161000 | 146500 | 0,91 |  |
|  | XP118BR | 456200 | 315400 | 0,69 | 0,72 |
|  |  | 125900 | 94750 | 0,75 |  |
